# The potential association between sedentary behaviors and risk of temporomandibular disorders: evidence from Mendelian randomization analysis

**DOI:** 10.22514/jofph.2024.042

**Published:** 2024-12-12

**Authors:** Junfei Zhu, Xuguang Yuan, Ye Zhang

**Affiliations:** ^1^Stomatology Center, China Japan Friendship Hospital, 100029 Beijing, China

**Keywords:** Sedentary behaviors, Temporomandibular disorders, Mendelian randomization

## Abstract

The role of sedentary behaviors in temporomandibular disorders (TMD) has not been thoroughly investigated. This study aims to investigate the potential association between sedentary behaviors and TMD using Mendelian randomization (MR) analysis. The MR method was employed to assess the causal association between sedentary behaviors and the risk of TMD. Genetic variants associated with sedentary behaviors, such as watching TV (Television), using computers and driving, were used as instrumental variables (IVs). MR analysis was performed using inverse variance-weighted (IVW) and weighted median methods, alongside MR-Egger regression to assess pleiotropy and statistical heterogeneity. Furthermore, leave-one-out analyses were conducted to assess whether any single SNP (single nucleotide polymorphism) or subset of SNPs influenced the results. Our analysis identified a significant association between driving time and the risk of temporomandibular disorders (IVW: OR (Odd ratio) = 2.797, 95% CI (Confidence interval) = 1.148–6.811, *p* = 0.024; weighted median OR = 4.271, 95% CI = 1.226–14.871, *p* = 0.023). In contrast, no significant associations were observed between time spent watching TV and using a computer and TMD risk. The robustness of the findings was confirmed through sensitivity analyses, including leave-one-out analysis. This study provides evidence of a potential genetic link between prolonged driving and TMD risk, suggesting that individuals frequently engaged in long-duration driving should be monitored for TMD symptoms. Further research is warranted to explore the complex interactions between sedentary behaviors and TMD, incorporating longitudinal and comprehensive assessments.

## 1. Introduction

Temporomandibular Disorders (TMD) is a broad term that includes a group of conditions affecting the temporomandibular joint (TMJ), the muscles responsible for jaw movement, and the associated structures [1]. Diagnosis of TMD is complex and can result in 12 distinct subtypes based on the category and severity of symptoms [2]. Common clinical symptoms of TMD include; pain in the jaw joint and surrounding tissues, limited jaw movement and clicking or popping sounds during jaw movement [3]. Approximately one-third of the adult population experiences one or more symptoms of TMD [4]. The onset of TMD can significantly impact an individual’s quality of life, leading to chronic pain, difficulty in eating and speaking and contributing to psychological distress [5].

Sedentary behaviors refer to activities that do not significantly increase energy expenditure above resting levels, typically defined as ≤1.5 metabolic equivalents (METs). These behaviors usually involve prolonged periods of inactivity and have been associated with adverse physical and systemic outcomes, including intervertebral disc disorders, bone fractures, obesity, cardiovascular disease and type 2 diabetes [6, 7, 8]. The rising prevalence of sedentary behaviors has raised significant public health concerns, contributing to substantial health and economic burdens [9]. Despite the well-documented health risks associated with sedentary behaviors, their potential association with TMD has not been thoroughly investigated.

Current research on TMD has primarily focused on mechanical factors, such as occlusal misalignment, bruxism (teeth grinding) and trauma, as well as psychosocial factors, including stress, anxiety and depression [10, 11]. These factors are critical in understanding the development and progression of TMD. The role of lifestyle behaviors, such as sedentary activities remain unexplored in TMD. For instance, work-related stress associated with computer use and the stress of driving in traffic can lead to increased tension in the masticatory muscles and promote oral parafunctional behaviors such as bruxism, potentially contributing to the first onset of TMD and the progression of complaints [12]. Given the widespread prevalence of sedentary behaviors and their known impact on musculoskeletal health [7], it is imperative to investigate their potential contribution to TMD.

Mendelian randomization (MR) is an analytical method of epidemiology that leverages genetic variants as instrumental variables to evaluate genetic relationships between exposures and outcomes. To date, studies examining the relationship between sedentary behaviors and TMD remain limited. Therefore, establishing this connection is important, as it could broaden the current understanding of TMD beyond traditional mechanical and psychosocial factors, enabling the identification of novel and modifiable risk factors. This study employed the MR method to investigate the potential causal association between sedentary behaviors (watching TV, using computers and driving) and TMD. We aim to identify and analyze genetic variants associated with these sedentary behaviors and TMD to infer potential relationships. By providing robust evidence of the association between sedentary behaviors and TMD, this study seeks to inform future research directions and public health interventions at preventing and managing TMD.

## 2. Materials and methods

### 2.1 Study design and data sources

The present study utilized the MR approach to evaluate the potential genetic association between sedentary behaviors (watching TV, using computers and driving) and TMD. Summary statistical data were obtained from genome-wide association studies (GWAS) from the UK Biobank (https://gwas.mrcieu.ac.uk/datasets/). Concisely, these datasets comprising 319,740, 261,987 and 224,813 individuals for time spent watching TV (ID: ukb-a-5), using a computer (ID: ukb-a-6) and driving (ID: ukb-a-7), respectively. Furthermore, outcome GWAS summary statistical data for TMD was retrieved from the MRC (Medical Research Council) Integrative Epidemiology Unit (IEU) database (https://gwas.mrcieu.ac.uk/datasets/finn-b-TEMPOROMANDIB/), which involves a sample size of 134,280, including 2730 cases and 131,550 controls.

### 2.2 Selection of genetic variants

For the instrumental variables (IVs), we selected single nucleotide polymorphisms (SNPs) associated with time spent watching TV and using a computer that met a stringent genome-wide significance threshold (*p *< 5 × 10^−8^). Less than 10 SNPs were identified under the *p* < 5 × 10^−8^ threshold for the genetic data of time spent driving. Therefore, a less stringent threshold of *p* < 5 × 10^−6^ was applied to ensure sufficient IVs for MR studies [13]. To minimize confounding from linkage disequilibrium (LD), LD clumping was employed to exclude SNPs close (*R*^2^ ≥ 0.001) to a more significantly associated SNP within a span of 1 megabase (Mb). Moreover, palindromic SNPs and those with uncertain allele frequencies were excluded before conducting MR analysis to prevent potential biases.

We calculated the *F*-statistic for every SNP to quantitatively assess the strength and precision of the association between each SNP and the exposure. The *F*-statistic was derived using the formula: *F* = (N − 2) × *R*^2^/(1 − *R*^2^), where *R*^2^ = 2 × ((beta.exposure)^2^) eaf.exposure × (1 − eaf.exposure). SNPs with an *F*-statistic lower than 10 were excluded to mitigate the risk of weak instrument bias, which could compromise the validity of the MR analysis.

### 2.3 Statistical analysis

The R software (version 4.3.2) was used for the data analysis. All reported estimates were calculated with a significance threshold of 0.05 or less. We used the inverse variance-weighted (IVW) method as the primary analytical approach to estimate the associations between sedentary behaviors and TMD risk. The IVW method assumes that all genetic variants are valid instruments. To ensure the integrity of our findings, we employed several supplementary MR methods, including MR-Egger regression, which estimates genetic associations even in the presence of pleiotropic effects, and the weighted median approach, which yields a consistent estimate if at least 50% of the information comes from valid instruments. Additionally, MR-Egger was used to test for pleiotropy by examining the intercept. The statistical heterogeneity among SNPs within the IVW and MR-Egger methods was evaluated using Cochran’s Q test, with heterogeneity deemed significant at a *p *< 0.05. We also implemented a leave-one-out analysis to determine if our results were driven by a single SNP or a subset of SNPs.

## 3. Results

Initially, 2985, 1621 and 714 SNPs were yielded for time spent watching TV, using the computer and driving, respectively. After LD clumping and *F*-statistics analyses, 59, 38 and 36 SNPs were retained for the MR statistical analysis. The details of the included SNPs are presented in **Supplementary Tables 1,2,3**.

### 3.1 Genetic association between the time spent watching TV and risk of TMD

The results of the IVW, MR-Egger and weighted median showed no significant genetic association between the time spent watching TV and risk of TMD: IVW (OR = 1.532, 95% CI = 0.711–3.301, *p* = 0.277), MR-Egger (OR = 0.101, 95% CI = 0.002–6.797, *p* = 0.290), and weighted median (OR = 1.031, 95% CI = 0.394–2.696, *p* = 0.950). The forest and scatter plots are presented in Figs. 1,2, respectively. Significant heterogeneity was observed for IVW (*p* = 0.021) and MR-Egger (*p* = 0.026). The MR-Egger method was also used to evaluate the pleiotropy of the IVs, revealing no significant difference between the MR-Egger intercept and the zero intercept of IVW (*p* = 0.203). A leave-one-out analysis was performed to assess the stability of the MR results by iteratively excluding each SNP. The overall error line remained stable throughout, indicating the robustness of the findings (Fig. 3).

**Fig. 1.** Forest plot of the genetic association between the time spent watching TV and risk of TMD. OR: Odds ratio; MR: Mendelian randomization.

**Fig. 2.** The scatter plot of the genetic association between the time spent watching TV and risk of TMD. MR: Mendelian randomization; TMD: Temporomandibular disorders; TV: Television; SNP: Single nucleotide polymorphisms.

**Fig. 3.** Leave-one-out sensitive analysis for the genetic association between the time spent watching TV and risk of TMD. MR: Mendelian randomization; TV: Television; TMD: Temporomandibular disorders.

### 3.2 Genetic association between the time spent using the computer on TMD

The results of IVW, MR-Egger and weighted median revealed no significant genetic association between the time spent using computer and risk of TMD: IVW (OR = 0.568, 95% CI = 0.239–1.349, *p* = 0.200), MR-Egger (OR = 0.039, 95% CI = 0.000–10.969, *p* = 0.267) and weighted median (OR = 0.777, 95% CI = 0.265–2.274,* p* = 0.645). The forest and scatter plots are presented in Figs. 4,5, respectively. Significant heterogeneity was observed for IVW (*p* = 0.037) and MR-Egger (*p* = 0.038). The MR-Egger method revealed no significant pleiotropy of the IVs: IVW (*p* = 0.352). The leave-one-out analysis confirmed the robustness of the findings (Fig. 6).

**Fig. 4.** Forest plot of the genetic association between the time spent using computer and risk of TMD. OR: Odds ratio; MR: Mendelian randomization.

.jpg

**Fig. 5.** The scatter plot of the genetic association between the time spent using computer and risk of TMD. MR: Mendelian randomization; TMD: Temporomandibular disorders; SNP: Single nucleotide polymorphisms.

.jpg

**Fig. 6.** Leave-one-out sensitive analysis for the genetic association between the time spent using computer and risk of TMD. MR: Mendelian randomization; TMD: Temporomandibular disorders.

.jpg

### 3.3 Genetic association between the time spent driving on TMD

Both the IVW and weighted median methods revealed significant associations between time spent driving and TMD risk, however, the result of MR-Egger was insignificant: IVW (OR = 2.797, 95% CI = 1.148–6.811, *p* = 0.024), MR-Egger (OR = 1.993, 95% CI = 0.068–58.663, *p* = 0.692), and weighted median (OR = 4.271, 95% CI = 1.226–14.871,* p* = 0.023). The forest and scatter plots are presented in Figs. 7,8, respectively. No significant heterogeneity was observed for IVW (*p* = 0.799) and MR-Egger (*p* = 0.762). Furthermore, the MR-Egger method revealed no significant pleiotropy of the IVs: IVW (*p* = 0.840). The leave-one-out analysis indicated the robustness of the findings (Fig. 9). The results of the MR analysis are presented in Table 1.

**Fig. 7.** Forest plot of the genetic association between the time spent driving and risk of TMD. OR: Odds ratio; MR: Mendelian randomization.

.jpg

**Fig. 8.** The scatter plot of the genetic association between the time spent driving and risk of TMD. MR: Mendelian randomization; TMD: Temporomandibular disorders; SNP: Single nucleotide polymorphisms.

.jpg

**Fig. 9.** Leave-one-out sensitive analysis for the genetic association between the time spent driving and risk of TMD. MR: Mendelian randomization; TMD: Temporomandibular disorders.

.jpg

**Table 1.** Genetic association between sedentary behaviors and TMD.

Exposure	Method	Numbers of SNPs	*b*	*p*	OR	95% CI
Time spent watching TV						
	MR-Egger	57.000	−2.292	0.290	0.101	0.002–6.797
	Weighted median		0.031	0.950	1.031	0.394–2.696
	Inverse variance weighted		0.426	0.277	1.532	0.711–3.301
Time spent using computer						
	MR-Egger	34.000	−3.254	0.645	0.777	0.000–10.989
	Weighted median		−0.253	0.645	0.777	0.265–2.274
	Inverse variance weighted		−0.565	0.200	0.568	0.239–1.349
Time spent driving						
	MR-Egger	34.000	0.689	0.692	1.993	0.068–58.663
	Weighted median		1.452	0.023	4.271	1.226–14.871
	Inverse variance weighted		1.028	0.024	2.797	1.148–6.811

*SNP: Single nucleotide polymorphisms; OR: Odds ratio; TV: Television; CI: Confidence interval; MR: Mendelian randomization.*

## 4. Discussion

This study explored the potential genetic association between sedentary and risk of TMD. The MR analysis revealed significant associations between driving time and an increased TMD risk. Specifically, the IVW (*p* = 0.024) and weighted median (*p* = 0.023) methods indicated that driving time is significantly associated with higher TMD risk. Conversely, no significant association was observed between time spent watching TV or using a computer and TMD risk across the primary MR methods.

TMD represents a group of conditions affecting the TMJ and the surrounding musculature. The etiology of TMD is multifactorial, encompassing various biopsychosocial factors [14]. Previous MR studies have indicated that the risk of developing TMD may be linked to short sleep duration, panic disorder, depression, rheumatoid arthritis and hyperthyroidism [15, 16, 17, 18, 19]. Evidence shows that the stomatognathic system and body posture influence each other, and postural changes could be a risk factor for TMD [20, 21, 22]. Observational studies indicate prolonged sedentary behavior negatively affects musculoskeletal health [23]. A lack of Physical activity is strongly linked to various chronic disorders, including cardiovascular disease, diabetes, as well as musculoskeletal disorders [24, 25]. However, direct evidence establishing a causality between sedentary behavior and TMD remains insufficient.

The observed associations between driving and TMD may be attributed to several underlying mechanisms. Firstly, prolonged periods of driving often require maintaining a static posture, which can lead to muscle fatigue and increased tension in the jaw and neck muscles [26]. This muscle tension may transfer to the TMJ, causing or worsening TMD symptoms. Additionally, the repetitive motions involved in driving can also contribute to cumulative microtrauma in the TMJ and surrounding muscles [27, 28]. This biomechanical stress could potentially exacerbate or contribute to the onset of TMD [29]. Moreover, high levels of stress and anxiety are commonly linked to driving. For example, the prolonged mental focus required and the stress of navigating traffic can exacerbate TMD symptoms through psychological pathways [30]. Chronic stress and anxiety are also known to contribute to bruxism (teeth grinding), which is another significant risk factor for TMD [31].

The lack of a significant association between watching TV or using a computer and TMD suggests that not all sedentary behaviors uniformly affect TMD risk. This distinction may be due to differing physical demands and postures associated with these activities. Traditionally, TV watching is a sedentary behavior associated with relaxing leisure time [32]. In contrast to the active engagement required during driving, watching TV and using a computer typically involve a more relaxed and passive posture.

A key strength of this study is the use of MR, which helps alleviate the influence of confounding factors, thereby providing more reliable evidence for potential genetic relationships. The large sample sizes included in the GWAS enhance the generalizability of our findings. Moreover, several sensitivity analyses were conducted, revealing no significant pleiotropy, and the robustness of the findings was confirmed by the leave-one-out analysis, thereby minimizing the potential for biased results.

However, several limitations should be considered. Firstly, significant heterogeneity was observed. Therefore, the relevant results should be interpreted with caution. Additionally, although the SNP data was derived from GWAS, critical details about the studied population, such as eligibility criteria and diagnostic standards for TMD were not provided. As a result, it was challenging to assess the quality of the studies that generated the IVs. Furthermore, several confounding factors, such as bruxism, were not accounted for in the present MR analysis due to the unavailability of SNP data in current GWAS studies. Therefore, further multivariable MR analyses are required to address these limitations. Moreover, although alterations in body posture and muscle fatigue in the masticatory muscles have been identified as potential risk factors for TMD [21, 33, 34], detailed data on the relationships between TMD and the postural changes, as well as muscle effects in other parts of the body, are limited. Therefore, further research in these areas is required to better understand the association between sedentary behaviors and TMD.

## 5. Conclusions

In conclusion, our study provides evidence for the potential genetic links between certain sedentary behavior and TMD. Prolonged driving may increase the risk of TMD. Individuals who often experience long periods of driving should pay attention to the occurrence of TMD. Future research should focus on longitudinal studies integrating detailed behavioral, biomechanical and psychological assessments to understand the complex interplay between sedentary behaviors and TMD.

## Supplementary Material


